# Selective Cytotoxicity of 1,3,4-Thiadiazolium Mesoionic Derivatives on Hepatocarcinoma Cells (HepG2)

**DOI:** 10.1371/journal.pone.0130046

**Published:** 2015-06-17

**Authors:** Gustavo Jabor Gozzi, Amanda do Rocio Andrade Pires, Glaucio Valdameri, Maria Eliane Merlin Rocha, Glaucia Regina Martinez, Guilhermina Rodrigues Noleto, Alexandra Acco, Carlos Eduardo Alves de Souza, Aurea Echevarria, Camilla Moretto dos Reis, Attilio Di Pietro, Sílvia Maria Suter Correia Cadena

**Affiliations:** 1 Departamento de Bioquímica e Biologia Molecular, Universidade Federal do Paraná, Curitiba, Paraná, Brazil; 2 Departamento de Farmacologia, Universidade Federal do Paraná, Curitiba, Paraná, Brazil; 3 Departamento de Química, Universidade Federal Rural do Rio de Janeiro, Rio de Janeiro, Brazil; 4 Equipe Labellisée Ligue 2014, BMSSI UMR 5086 CNRS/Université Lyon 1, IBCP, Lyon, France; University of Navarra School of Medicine and Center for Applied Medical Research (CIMA), SPAIN

## Abstract

In this work, we evaluated the cytotoxicity of mesoionic 4-phenyl-5-(2-Y, 4-X or 4-X-cinnamoyl)-1,3,4-thiadiazolium-2-phenylamine chloride derivatives (MI-J: X=OH, Y=H; MI-D: X=NO_2_, Y=H; MI-4F: X=F, Y=H; MI-2,4diF: X=Y=F) on human hepatocellular carcinoma (HepG2), and non-tumor cells (rat hepatocytes) for comparison. MI-J, M-4F and MI-2,4diF reduced HepG2 viability by ~ 50% at 25 μM after 24-h treatment, whereas MI-D required a 50 μM concentration, as shown by 3-(4,5-dimethythiazol-2-yl)-2,5-diphenyltetrazolium bromide assays. The cytotoxicity was confirmed with lactate dehydrogenase assay, of which activity was increased by 55, 24 and 16% for MI-J, MI-4F and MI-2,4diF respectively (at 25 μM after 24 h). To identify the death pathway related to cytotoxicity, the HepG2 cells treated by mesoionic compounds were labeled with both annexin V and PI, and analyzed by flow cytometry. All compounds increased the number of doubly-stained cells at 25 μM after 24 h: by 76% for MI-J, 25% for MI-4F and MI-2,4diF, and 11% for MI-D. It was also verified that increased DNA fragmentation occurred upon MI-J, MI-4F and MI-2,4diF treatments (by 12%, 9% and 8%, respectively, at 25 μM after 24 h). These compounds were only weakly, or not at all, transported by the main multidrug transporters, P-glycoprotein, ABCG2 and MRP1, and were able to slightly inhibit their drug-transport activity. It may be concluded that 1,3,4-thiadiazolium compounds, especially the hydroxy derivative MI-J, constitute promising candidates for future investigations on *in-vivo* treatment of hepatocellular carcinoma.

## Introduction

**Fig 1:**
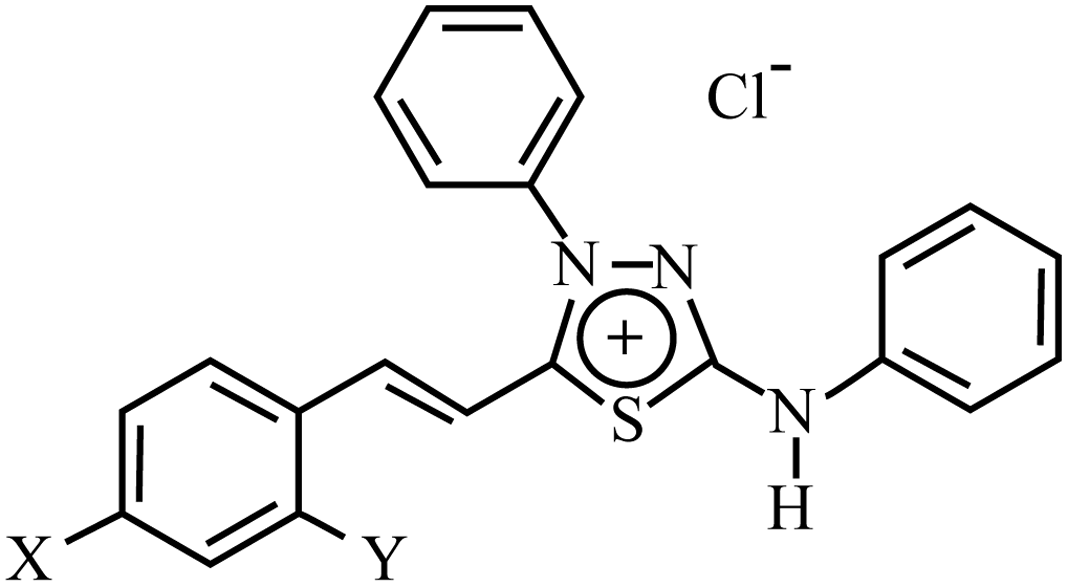
Chemical structure of the 4-phenyl-5-(2-Y-4-X-cinnamoyl)-1,3,4-thiadiazolium-2-phenylamine chloride derivatives: MI-D (X = NO_2_; Y = H), MI-J (X = OH; Y = H), MI-4F (X = F; Y = H), and MI-2,4diF (X = Y = F).

Liver cancer is the third most common cause of cancer-related death worldwide [1, 2] Hepatocellular carcinoma (HCC), specifically, represents the major histological subtype among primary liver cancer [1, 3], being one of the most prevalent malignant tumor worldwide [1]. Surgical resection and transplantation still remain the first choice of HCC treatment with potential cure, but this procedure must be used only in patients with early stages of HCC [4, 5]. Unfortunately, diagnosis often occurs in HCC advanced stages [2], and there is then only one—drug approved by Food and Drug Administration (FDA) that can be used as a systemic therapeutic agent for HCC treatment [6]. Other drug-based therapies have promisingly emerged as alternatives for early- and advanced- HCC treatment, which has motivated the research of new compounds to be used in patients who are not candidates to surgical treatment [5–7]. The high toxicity of drugs toward non-tumoral cells and the resistance to treatment constitute great problems in present chemotherapy [8, 9]. Drug toxicity usually limits the concentration usable for the treatments, as well as the frequency of administrations, further affecting curing efficiency [10]. Additionally, tumor cells may become resistant to drugs through different mechanisms. The most notable one is the overexpression of ATP-binding cassette transporters, such as P-glycoprotein (Pgp) [11], multidrug resistance protein 1 (MRP1) [12] and breast cancer resistant protein (ABCG2) [13], which efflux several types of drugs with unrelated structures and mechanisms [14]. This feature is a main obstacle to effectiveness of chemotherapy against HCC [15]. Indeed, several studies have demonstrated a relationship between overexpression of these efflux pumps and either poor prognosis or aggressive tumor phenotype in patients with HCC [16–18]. Mesoionic compounds with a 1,3,4-thiadiazolium ring have shown important biological activities as antibiotic [19], antiparasitic [20], antiviral [21], anticonvulsant [22], antidepressant [23], antioxidant [24], analgesic, antiinflammatory [25] and antitumoral [26] agents. We have specifically studied several 1,3,4-thiadiazolium-2-phenylamine chlorides mesoionic derivatives, only differing through the substituents of the cinnamoyl ring: MI-D, X = NO_2_; MI-J, X = OH; MI-4F, X = F; MI-2,4diF, X = Y = F (Fig 1). Some of them have demonstrated antitumoral effects against carcinoma, sarcoma [27] and melanoma [26, 28] tumors *in vivo*, and cytotoxic activities against several types of tumor cells have been described *in vitro* [29]. Otherwise, it has been shown that these derivatives promote functional and structural alterations in isolated rat liver mitochondria, up to different degrees depending on cinnamoyl ring substitution [30–33]. We evaluated the cytotoxicity of MI-D, MI-J, MI-4F and MI-2,4diF on human hepatocellular carcinoma cells (HepG2), and primary rat hepatocytes as a non-tumoral model, and their effects on the multidrug resistance proteins Pgp, ABCG2 and MRP1. It was found that these compounds might represent new alternatives for HCC chemotherapeutic treatments, overcoming important limiting problems such as drug resistance and toxicity toward non-tumoral cells.

## Materials and Methods

### 2.1 Chemicals

High-glucose Dulbecco’s modified Eagle’s medium (DMEM) was obtained from Cultilab (Campinas, Brazil) and fetal bovine serum (FBS) was purchased from Gibco. Dimethylsulfoxide (DMSO) was obtained from Merck (São Paulo, SP, Brazil). Annexin V Apoptosis Detection Kit was purchased from BD Bioscience (São Paulo, SP, Brazil). Lactate dehydrogenase (LDH) detection kit (Liquiform) was obtained from Labtest (Lagoa Santa, MG, Brazil). Bovine serum albumin (BSA), 3-(4,5-dimethythiazol-2-yl)-2,5-diphenyltetrazolium bromide (MTT) and 4-(2-hydroxyethyl)-1-piperazine ethanesulfonic acid (HEPES) were purchased from Sigma. All other reagents were commercial products of the highest available purity grade. The mesoionic derivatives, MI-D (4-phenyl-5-(4-nitrocinnamoyl)-1,3,4-thiadiazolium-2-phenylamine chloride), MI-J (4-phenyl-5-(4-hydroxycinnamoyl)-1,3,4-thiadiazolium-2-phenylamine chloride), MI-4F (4-phenyl-5-(4-fluorocinnamoyl)-1,3,4-thiadiazolium-2-phenylamine chloride), MI-2,4diF (4-phenyl-5-(2,4-fluorocinnamoyl)-1,3,4-thiadiazolium-2-phenylamine chloride), were synthesized by the Department of Chemistry of the Federal Rural University of Rio de Janeiro, Brazil, and their structures were confirmed by ^1^H NMR,^13^C NMR and mass spectrometry [34]. For this study, the derivatives were dissolved in DMSO and then further diluted with the assay medium. Controls with DMSO (maximal 0.7%, v/v) were carried out in each assay.

### 2.2 HepG2 cell culture

The human hepatocarcinoma HepG2 cell line (from the American Type Culture Collection—ATCC) was maintained in high-glucose DMEM supplemented with 10% FBS, 100 UI/mL penicillin G and 100 μg/mL streptomycin, 20 mmol/L 4-(2-hydroxyethyl)-1-piperazine ethanesulfonic acid (HEPES), adjusted to pH 7.4 with 1 mol/L sodium bicarbonate. HepG2 cells were grown in poly-L-lysine-coated flasks at 37°C, 5% CO_2_ under controlled humidity. Sub-culturing was performed at approximately 48 h intervals, and cell growth was monitored with an Olympus inverted microscope.

### 2.3 Primary culture of rat hepatocytes

#### 2.3.1 Animals

Male Wistar rats (180–200 g) were obtained from the Central Animal House of the Federal University of Paraná (PR, Brazil). The animals received a standard laboratory diet (Purina) and tap water. This study was carried out in strict accordance with the recommendations in the Guide for the Care and Use of Laboratory Animals of the National Institutes of Health. The protocol was approved by the Committee on the Ethics of Animal Experiments of the University Federal of Paraná (Permit Number: 548). All surgery was performed under ketamine/xylazine anesthesia, and all efforts were made to minimize suffering.

#### 2.3.2 Isolation and culture of hepatocytes

The hepatocytes were obtained by monovascular liver perfusion of Wistar rats, as described previously by [35, 36] with some modifications. The male rats were weighed and anesthetized intraperitoneally with a mixture of ketamine (60 mg/kg) and xylazine (7.5 mg/kg). Following laparotomy, 100 μL of sodium heparin (5000 U/mL) were injected into the abdominal cava vein. The portal and thoracic cava veins were cannulated, and the liver was perfused for 10–15 min with Krebs solution (2.4 mol/L NaCl, 96 mmol/L KCl, 24 mmol/L KH_2_PO_4_, 24 mmol/L MgSO_4_, 480 mmol/L NaHCO_3_ and 1 mol/L HEPES buffer, pH 7.4) containing 1.3 mol/L CaCl_2_, 20 mg collagenase (types IA and IV) and carbogen (95% O_2_:5% CO_2_). The liver was excised, and the cells were released by mechanical action, filtered through 50-μm nylon membranes and centrifuged at 400 rpm for 5 min at 4°C. Subsequently, the cells were centrifuged four times with Krebs solution supplemented with 20% BSA and treated with carbogen. The cells were suspended in high-glucose DMEM supplemented with FBS (10%), insulin (100 nmol/L), glucagon (10 nmol/L), epidermal growth factor (10 ng/mL), dexamethasone (50 nmol/L), penicillin (100 U/mL) and streptomycin (100 ng/mL). Cell viability was determined using the Trypan blue (0.4%, w/v) exclusion method as previously described by Philips [37]. Only the cell suspensions with viabilities higher than 80% were plated (1x10^6^ cells/plate on a 60 mm plate) and cultured for further experiments. For 4h after plating, the medium was replaced by Hepatozyme with or without mesoionic compounds. Considering some delays during the isolation procedure and the time required for further assays (e.g. viability assays), the time of treatment was from 18 to 24h. It is important to remark that no differences in the results were observed during this interval.

### 2.4 Culture of multiple drugs resistant cells

The human embryonic kidney (HEK293) cells stably transfected with *ABCG2* (HEK293*ABCG2*) [38] or *MRP1* (HEK293*ABCC1*), and their respective parental HEK293 (wild-type) or HEK293*pcDNA5* (empty-vector) cells, were maintained at 37°C (5% CO_2_) in high-glucose DMEM medium, supplemented with 10% fetal bovine serum, 1% penicilin/streptomycin. The mouse embryonic fibroblasts, of either wild-type (NIH3T3) or overexpressing Pgp (NIH3T3*ABCB1*) [39], were maintained under the same conditions. The cell culture media were drug supplemented with either 0.75 mg/mL G418 (HEK293*ABCG2*), 200 μg/mL hygromycin B (HEK293*pcDNA5* and HEK293*ABCC1*) or 60 ng/mL colchicin (NIH3T3*ABCB1*).

### 2.5 Cell viability assays

#### 2.5.1 MTT reduction

HepG2 cells were seeded at a density of 1×10^4^ cells/well into 96-well culture plates. After 24 h, the cells were treated with mesoionic compounds at concentrations of 5, 25 and 50 μM for 24 h. Hepatocytes were seeded at 1×10^6^ cells/plate in 60-mm plates, and treated with compounds at 25 μM up to 18–24 h. Cell viability was evaluated through the MTT assay [40, 41], and the absorbance was determined at 550 nm. The results were expressed as a percentage of viable cells in comparison to the control (taken as 100%). All HEK293 cells were seeded at a density of 1 × 10^4^ cells/well into 96-well culture plates, and incubated for 24 h at 37°C in 5% CO_2_. NIH−3T3 and NIH−3T3*ABCB1* cells were seeded at a density of 3.5 × 10^3^ and 5.0 x 10^3^ cells/well, respectively, and maintained under the same conditions before treatment. The cells were treated with mesoionic derivatives for 72 h; then, 20 μL of MTT solution (5 mg/mL) were added to each well and incubated for 4 h at 37°C. The culture medium was discarded, and 100 μL of a DMSO:ethanol (1:1) solution was added into each well and mixed by gently shaking for 10 min. Absorbance was measured in a microplate reader at 570 nm, from which the value measured at 690 nm was subtracted.

#### 2.5.2 Lactate dehydrogenase release

HepG2 and hepatocytes cells were plated and treated as described in the item 2.4.1. Aliquots (50 μL) of culture medium were centrifuged at 1000 rpm for 5 min, and LDH activity was measured by monitoring the decrease of NADH at 340 nm, with the LDH kit assay, according to manufacturer instructions.

#### 2.5.3 Annexin-V and propidium iodide staining

HepG2 cells were seeded at a density of 1×10^6^ cells in 60-mm plates, and treated with mesoionic derivatives at 25 μM for 24 h. At the end of exposure, cells were collected by trypsinization, centrifuged at 300x*g* for 5 min at 4°C and suspended in 500 μL of binding buffer (10 mM Hepes, pH 7.4, 150 mM NaCl, 5mM KCl, 1 mM MgCl_2_ and 1.8 mM CaCl_2_). Aliquots (100 μl) of the cell suspension were incubated with 5 μl of the reagent mixture containing annexin V-FITC conjugate (BD Pharmingen) and 10 μl of propidium iodide (PI) (50 μg/mL) for 15 min at 25°C. After incubation, 400 μl of binding buffer were added and the cells were analyzed by flow cytometry [42]. Positive controls were separately stained with Annexin V alone (channel FL-1), PI alone (channel FL-2), and both markers, for compensation settings of the two signals. Flow cytometric analysis was carried out on a FACSCalibur flow cytometer (BD Biosciences Pharmingen, San Diego, CA, USA). In each sample, 10.000 events were recorded and analysis was performed using the WinMDI 2.9 software. Three independent experiments were performed for each treatment condition. Hepatocytes were seeded at 1×10^6^ cells/plate in 60-mm plates, and treated with compounds at 25 μM up to 18–24 h. The culture medium was then replaced by the binding buffer containing 5 μl of annexin-V FITC and 0.8 mg/mL of PI. Cells were analyzed by fluorescence microscopy (AXIOVERT 40CSFL) with a 10X objective, in either absence (visible) or presence of annexin filter—FL1: 515–530 nm or PI-FL2: 560–580 nm.

### 2.6 Morphology assays

HepG2 cells (1x10^5^ cells/well) were seeded in 24-well plates with glass slides on the bottom, and incubated in a humidified incubator with 5% CO_2_ and at 37°C for 24 h. After adhesion, the medium was replaced by new medium with or without mesoionic derivatives at 5 μM for 3 h. The cells were fixed with Bouin solution (formaldehyde at 4% (v/v):saturated picric acid:glacial acetic acid, 4:15:1) for 5 min at ambient temperature. Then, the cells were washed with ultrapure water, and stained with hematoxylin and eosin. They were dehydrated with acetone and xylol solutions, and assembled with Entelan (Merck). The morphological alterations were viewed in Bel Fotonics microscope with 40 X and 100 X magnification, and the images were captured by a photographic camera Sony Cyber-Shot at 13.5 mega pixels. Hepatocytes were seeded at 1×10^6^ cells/plate in 60-mm and treated with compounds at 25 μM up to 18–24 h. After exposure, the morphology was analyzed by optical microscopy (AXIOVERT 40CSFL) with a 20X objective.

### 2.7 DNA fragmentation

The fragmented DNA content was determined by flow cytometry using PI [43–45]. For these assays, 1x10^6^ cells were dispensed in 60-mm plates, and incubated for 24 h for adhesion at 37°C in 5% CO_2_. The culture medium was then replaced by fresh medium without (control) or with 25 μM derivatives, or the corresponding volumes of DMSO, and further incubated for 6 and 24 h. After incubation, the culture medium and cells were collected in a Falcon tube by tripsinization, and the samples were centrifuged at 2000 rpm for 5 min. The precipitate was suspended in phosphate buffered saline (PBS) and centrifuged again under the same conditions. The cells were suspended in 0.3 mL of a solution composed of 50 μg/mL of propidium iodide and 0.1% Triton X -100 in PBS. After labeling, the cells were kept in the dark and analyzed by flow cytometry with the FACSCalibur (BD) apparatus using Cell Quest program. The data acquisition was done using the FL2 filter (yellow fluorescence), and analyzed as histograms (FL2 *versus* number of events). The number of cells in each phase of the cell cycle was expressed as a percentage of total events (10.000 cells). Histograms were analyzed using the WinMDI 2.9 software.

### 2.8 Protein concentration assay

Protein concentration was determined by the method of Bradford [46] using bovine serum albumin BSA as a standard.

### 2.9 Inhibition of drug efflux in multiple drug resistant cells

HEK293*ABCG2* cells were seeded at a density of 1.0×10^5^ cells/well into 24-well culture plates. After 72-h incubation, they were exposed to 5 μM mitoxantrone for 30 min at 37°C, in the presence or absence of each derivative, and then washed with PBS and trypsinized. The intracellular fluorescence was monitored with a FACSCalibur cytometer (Becton Dickinson), using the FL4 channel and at least 10,000 events were collected. The percentage of inhibition was calculated relatively to 1 μM Ko143 which produced a complete inhibition. NIH−3T3*ABCB1* were seeded at a density of 6 × 10^4^ cells/well into 24-well culture plates and incubated for 48 h at 37°C, whereas HEK293 cells transfected with *ABCC1* were seeded at 2.5 × 10^5^ cells/well for 72 h. The cells were respectively exposed to rhodamine 123 (0.5 μM) or calcein-AM (0.2 μM) for 30 min at 37°C, in the presence or absence of each derivative, then washed with PBS and trypsinized. The intracellular fluorescence was monitored using the FL1 channel. The inhibition was measured relatively to 5 μM GF120918 or 35 μM verapamil, respectively, producing complete inhibitions. The percentage of inhibition was calculated by using the following equation:

$$\%\;inhibition\;=\;(C-M)\;/\;(C_{ev}-M)\text{ x }100,$$

where C corresponds to the intracellular fluorescence of resistant cells in the presence of compounds and fluorescent substrate, M to the intracellular fluorescence of resistant cells with the fluorescent substrate alone, and C_ev_ corresponds to the intracellular fluorescence of cells inhibited with standard inhibitor in the presence of fluorescent substrate.

### 2.10 Statistical Analysis

Results were expressed as mean ± standard deviation, and subjected to analysis of variance (ANOVA) and Tukey test for comparison of means. A *P*-value lower than 0.05 was considered significant. All analyses and graphs were performed using GraphPad Prism Software version 6.0.

## Results

### 3.1 Cytotoxicity of 1,3,4-thiadiazolium derivatives on HepG2 and rat hepatocytes

**Fig 2:**
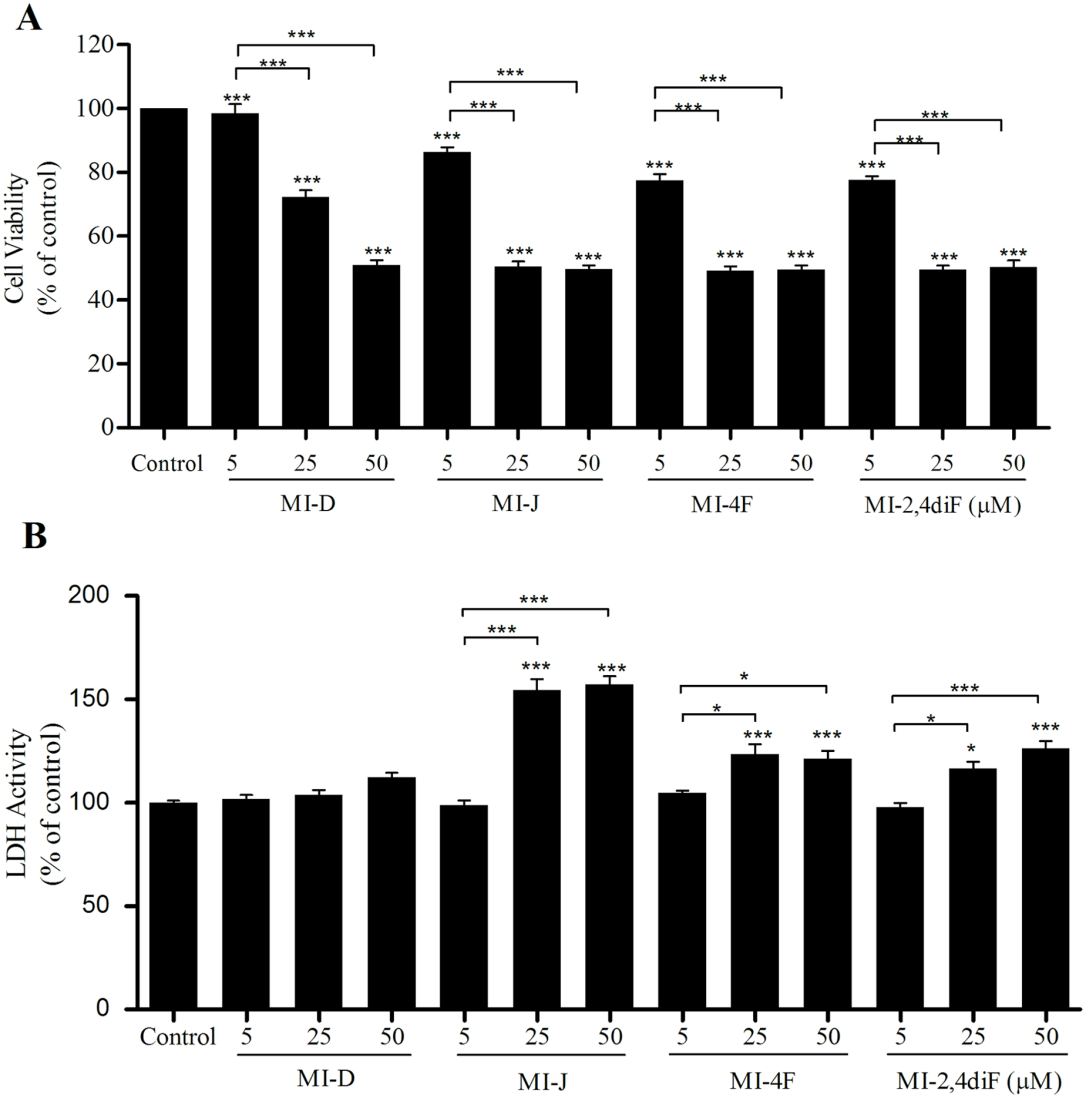
Cytotoxic effects of 1,3,4-thiadiazolium derivatives on HepG2 cells. A. MTT assay (the experimental conditions are described in the Materials and Methods section 2.5.1). The cells were seeded with or without 1,3,4-thiadiazolium derivatives at 5, 25 or 50 μM for 24 h. The results were expressed as % of viability in comparison to control. B. LDH release assay (the experimental conditions are described in the Materials and Methods section 2.5.2). Under the same treatment conditions, as described above, LDH activity was measured in supernatants. Data represent means of four different experiments in quadruplicate The results were expressed as % of viability in comparison to control. * and *** denote values significantly different from the control or between the different treatments at *P*< 0.05 and *P*< 0.0001, respectively.

**Fig 3:**
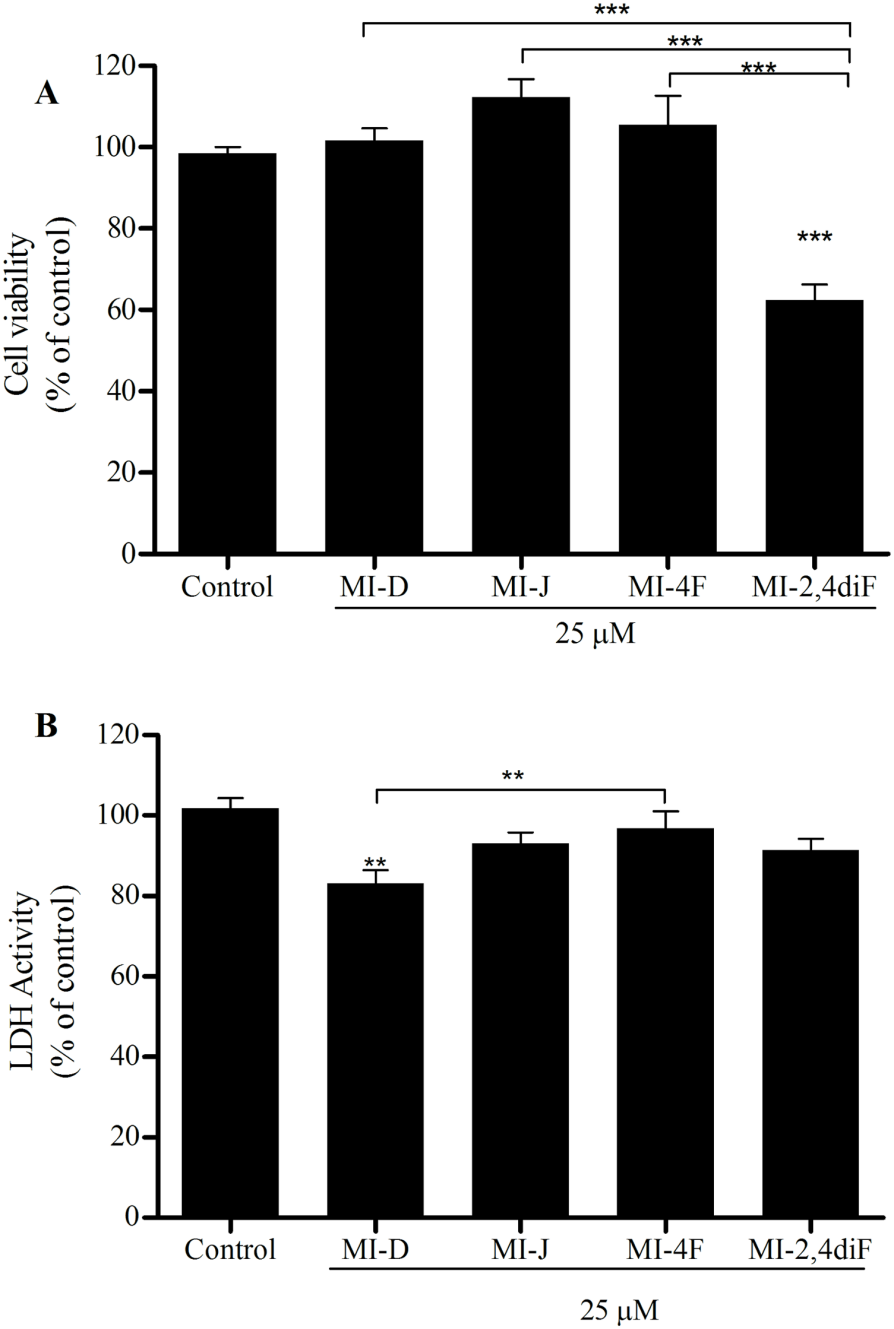
Cytotoxic effects of 1,3,4-thiadiazolium derivatives on hepatocytes. A. MTT assay (the experimental conditions are described in the Materials and Methods section 2.5.1) The cells were seeded with or without 1,3,4-thiadiazolium derivatives at 25 for 18–24 h. The results were expressed as % of viability in comparison to control. B. LDH release assay (the experimental conditions are described in the Materials and Methods section 2.5.2). Under the same treatment conditions described above, LDH activity was measured in the supernatants. Data represent means of four different experiments in quadruplicate. The results were expressed as % of viability in comparison to control. ** and *** denotes values significantly different from the control or between the different treatments at *P*< 0.01 and *P*< 0.0001, respectively.

The viability of HepG2 cells was determined after 24 h of treatment with derivatives at 5, 25 and 50 μM, by both MTT and LDH-release assays. As observed in Fig 2, upper panel, MI-J, MI-4F and MI-2,4diF reduced HepG2 cells viability by about 50% at 25 μM when analyzed by MTT. MI-D only reduced by 28% the cell viability, requiring 50 μM to reach 50%. The results of the LDH-release assay (Fig 2, lower panel) also demonstrated the reduction of cell viability by MI-J, MI-4F and MI-2,4diF treatments. The enzymatic activity of the culture medium was increased by 55, 24 and 16%, respectively, for MI-J, MI-4F and MI-2,4diF at 25 μM, in comparison to controls without mesoionic derivative. MI-D, on the contrary, did not significantly affect the LDH activity. The viability of primary hepatocytes was also determined in order to verify the selectivity of derivatives for tumor cells. As observed in Fig 3, no cytotoxicity was observed in MTT assays (upper panel), except for MI-2,4diF producing a 36% effect (at 25 μM for 18–24 h). However, no increase in LDH activity was observed with any derivative (lower panel). Interestingly, MI-D induced a reduction of LDH activity (~ 17% at 25 μM).

### 3.2 Apoptosis induction by 1,3,4-thiadiazolium derivatives in HepG2 cancer cells but not in control hepatocytes

**Fig 4:**
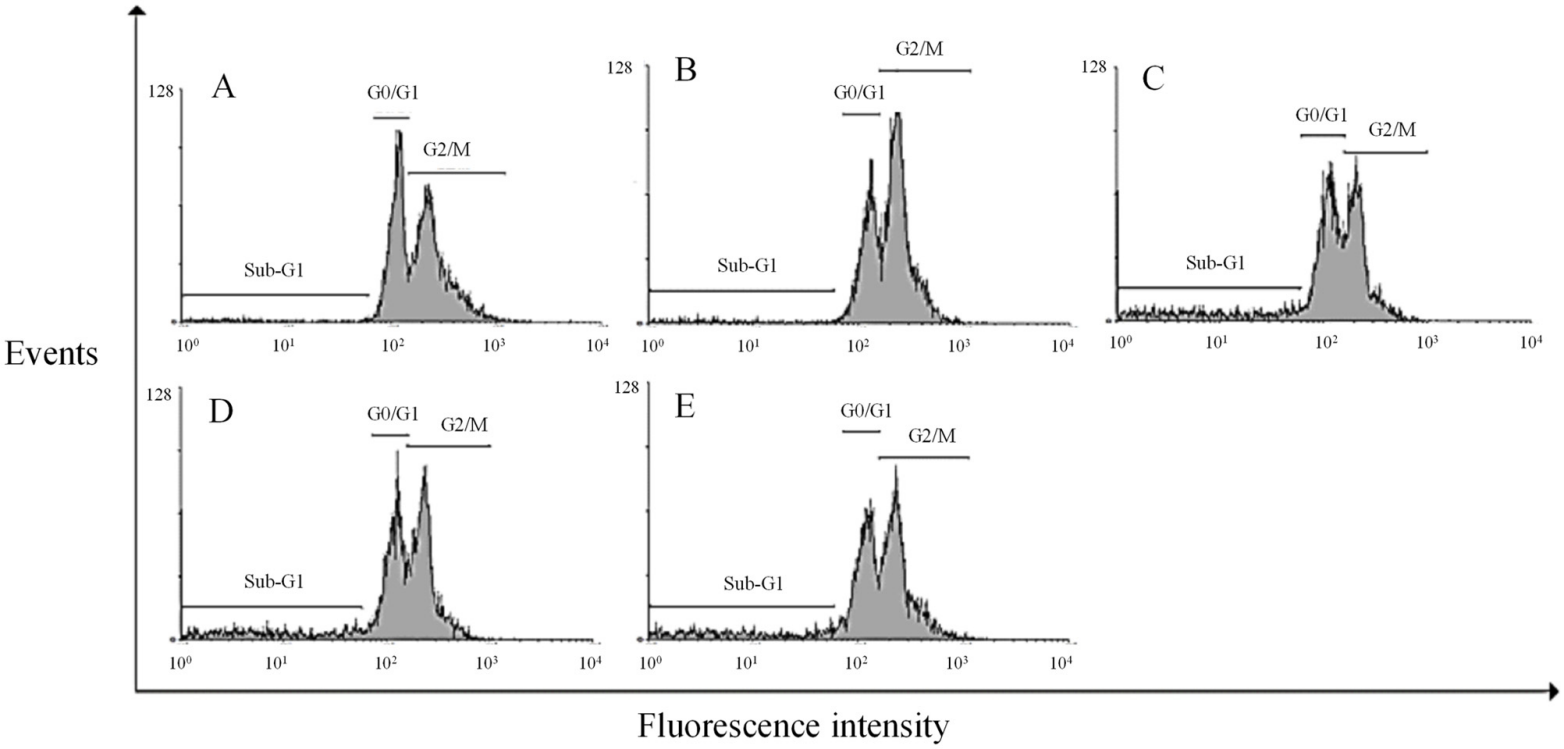
DNA fragmentation in HepG2 cells, as induced by 1,3,4-thiadiazolium derivatives (the experimental conditions are described in the Materials and Methods section 2.7). The cells were seeded with or without 1,3,4-thiadiazolium derivatives at 25 μM for 24 h. For each sample, 10.000 events were analyzed by flow cytometry using FL2 filter. (A) control, (B) MI-D, (C) MI-J, (D) MI-4F and (E) MI-2,4diF. The histograms represent three different experiments in triplicate.

**Fig 5:**
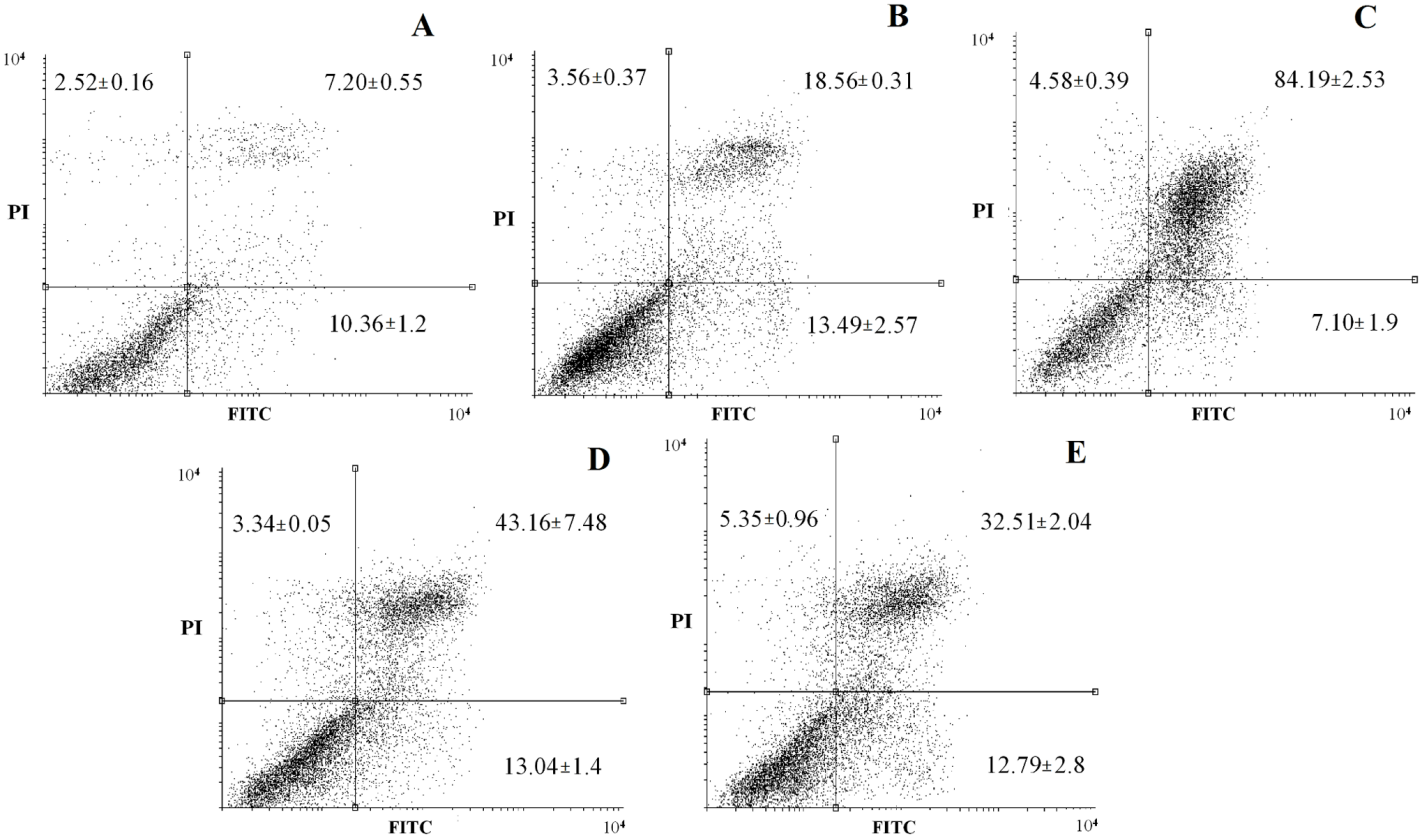
Annexin V-FITC and propidium iodide staining of HepG2 treated with 1,3,4-thiadiazolium derivatives (the experimental conditions are described in the Materials and Methods section 2.5.3). The cells were seeded with or without 1,3,4-thiadiazolium derivatives at 25 μM for 18–24 h. Then, the cells were collected with trypsin and 10.000 events were analyzed by flow cytometry by FL2 and FL1 filters. (A) control, (B) MI-D, (C) MI-J, (D) MI-4F and (E) MI-2,4diF. The figures show representative dot-plot with the different cell populations: left-bottom = labeled cells; left-top = PI labeled; right-top = doubly labeled; right-bottom = annexin V labeled. The results were expressed as mean ± SD of three independents experiments.

**Fig 6:**
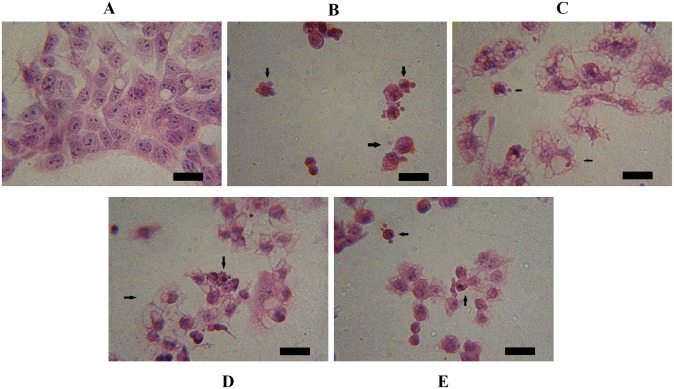
Effects of 1,3,4-thiadiazolium derivatives on HepG2 cell morphology (the experimental conditions are described in the Materials and Methods section 2.6). The cells were seeded with or without 1,3,4-thiadiazolium derivatives at 5 μM for 3 h. The images were captured with a 100X magnification; they correspond to control (A), MI-D (B), MI-J (C), MI-4F (D) and MI-2,4diF (E). The scale is indicated by black bars representing 0.02 mm. The arrows show morphological modifications as blebs, increased volume and vacuolization. The photographs represent three different experiments in triplicate.

**Fig 7:**
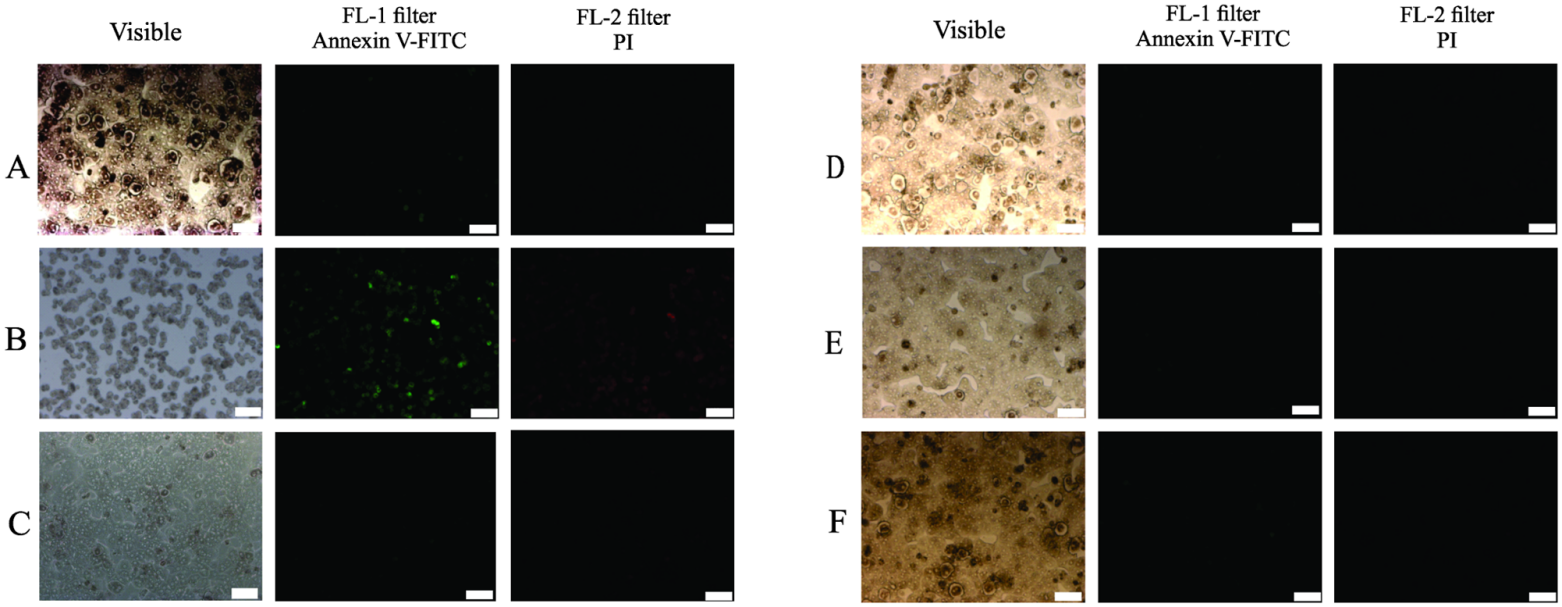
Annexin V-FITC and propidium iodide staining of hepatocytes treated by 1,3,4-thiadizolium derivatives (the experimental conditions are described in the Materials and Methods section 2.5.3). Hepatocytes were incubated with derivatives at 25 μM for 20 h. The images (10X magnification) were captured with an AXIOVERT 40CSFL fluorescence microscope. The scale is indicated by white bars representing 100 μm. The annexin V-FITC-positive cells are stained in green, and the PI-positive cells in red. The images represent (A) control (untreated cells), (B) ASA positive control at 20 mM, (C) MI-D, (D) MI-J, (E) MI-4F and (F) MI-2,4diF. The figures represent three different experiments in triplicate.

**Fig 8:**
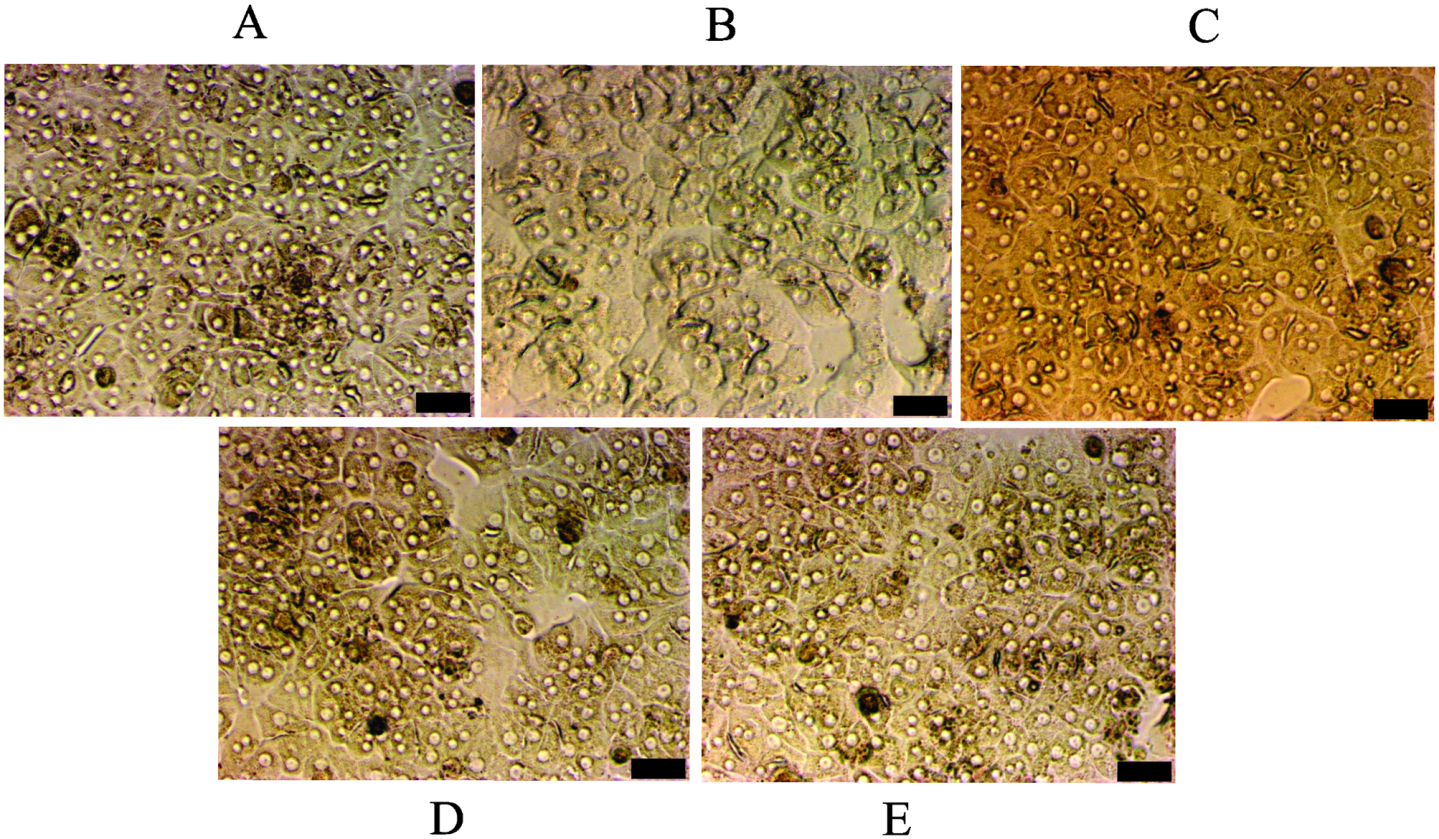
Effects of 1,3,4-thiadiazolium derivatives on hepatocytes morphology (the experimental conditions are described in the Materials and Methods section 2.6). Hepatocytes were incubated with the derivatives at 25 μM for 24 h. The images were obtained using inverted microscope. A: control (untreated cells); B-E: treatments by MI-D, MI-J, MI-4F and MI-2,4diF, respectively. The scale is indicated by black bars representing 50 μm. The photographs represent three different experiments in triplicate.

Considering the significant toxicity of the derivatives on HepG2 cells (Fig 2), we evaluated the induction of apoptosis in these cells by DNA fragmentation, a key event of cells undergoing apoptosis [47]. After 24 h of treatment with MI-J, MI-4F and MI-2,4diF (Fig 4C–4E), approximate increases of 12%, 9% and 8%, respectively, were observed, as evidenced by the higher number of cells in sub-G1 region. MI-D under the same conditions did not promote any significant alteration in DNA fragmentation, but increased the number of cells in G2/M phase (Fig 4B). The G1/G0 and G2/M phases were not significantly changed by the other derivatives. To further investigate the induction of apoptosis by mesoionic derivatives, HepG2 cells were simultaneously stained with FITC-conjugated annexin V and PI, and analyzed by flow cytometry (Fig 5). All compounds (at 25 μM for 24 h) increased the number of doubly-stained cells in comparison to control, reaching up to 76% for MI-J, 36% and 25% for MI-4F and MI-2,4diF, while a lower value of 11% was observed for MI-D. In addition, MI-J and MI-2,4diF promoted a slight increase (around 2.4%) in the number of PI-labeled cells. Since the differentiation between apoptosis and necrosis was not possible with such an assay, short incubation time (3 h) and reduced concentration (5 μM) were used for morphological analyzes [47]. Apoptotic bodies (blebs) were observed, and loss of cellular organization in monolayer was elicited for all compounds even at low concentration (Fig 6). Other characteristics of apoptosis induction, such as vacuolization, cellular shrinkage (with MI-D, MI-J and MI-4F) and nuclear pyknosis (with MI-4F and MI-2,4diF), were also observed. All together, these results suggest that apoptosis may be the death pathway induced by 1,3,4-thiadizolium derivatives on HepG2 cells. Cultured hepatocytes were also doubly stained with FITC-conjugated annexin V and PI, and analyzed by fluorescence microscopy (Fig 7), but no increase in annexin-FITC and PI labeling was observed for any compound (at 25 μM for 18–24 h) when compared to treatment with acetylsalicylic acid (at 20 mM for 18–24 h), used as a positive control [48]. The hepatocytes morphology was also verified: no alterations in normal characteristics of primary hepatocytes, as cubic form, monolayer organization and multinucleation was observed, supporting previous results suggesting that these derivatives slightly or not at all affected hepatocytes viability (Fig 8).

### 3.3 Effects of 1,3,4-thiadiazolium derivatives on multiple drugs resistant (MDR) cells

The effects of 1,3,4-thiadiazolium derivatives were checked on cells overexpressing multidrug ABC transporters, in order to establish their capacity to inhibit the transport of substrate drugs and/or to be transported themselves. Flow cytometry was used to analyze their capacity to induce accumulation of fluorescent substrates. Table 1 shows that, among the different mesoionic derivatives, only MI-J was able to significantly inhibit the Pgp-mediated efflux of rhodamine 123, with 13% inhibition (at 25 μM upon 30-min incubation) as compared to the control which was fully inhibited by 5 μM elacridar. The ability of the derivatives to be transported by Pgp was evaluated by the resistance ratio (RR), which was obtained by dividing the IG_50_ values estimated from survival MTT assays for transfected cells overexpressing the multidrug transporter and its parental, sensitive line, with RR values > 1 suggesting transporter-mediated efflux [49]. Except for MI-J which was not to be transported by Pgp, with a RR value even <1, the other derivatives indeed appeared to be transported, with high RR values of 5.2 for MI-2,4diF, and in the range 2.4–2.8 for MI-D and MI-4F.

**Table 1:** Effects of 1,3,4-thiadiazolium derivatives on MDR cell parameters.

	Pgp				ABCG2				MRP1			
	EC_50_	%inhibition	^a^IG_50_	^b^IG_50_	EC_50_	%inhibition	^a^IG_50_	^b^IG_50_	EC_50_	%inhibition	^a^IG_50_	^b^IG_50_
MI-D	n.d.	1.0	24.9 ± 5.7	9.0 ± 0.2	24.3 ± 9.6	36.7 ± 14.4	7.0 ± 0.1	8.9 ± 1.0	n.d.	8.1 ± 4.2	9.4 ± 0.5	7.5 ± 1.0
MI-J	n.d.	13.6 ± 6.3	9.8 ± 2.0	10.6 ± 0.5	8.5 ± 4.8	21.5 ± 12.1	7.9 ± 0.1	6.0 ± 0.1	n.d.	36.2 ± 24.7	4.9 ± 0.2	5.8 ± 0.1
MI-4F	n.d.	0	3.3 ± 0.1	1.4 ± 0.1	n.d.	11.7 ± 3.9	4.7 ± 0.2	5.7 ± 0.3	n.d.	10.1 ± 6.6	6.2 ± 0.1	6.0 ± 0.1
MI-2,4diF	n.d.	0.15	5.2 ± 0.2	1.0 ± 0.1	n.d.	17.7 ± 12.6	3.3 ± 0.3	4.6 ± 0.9	n.d.	21.0 ± 13.1	4.7 ± 0.5	5.5 ± 0.5

The experimental conditions are described in the Materials and Methods, sections 2.5.1 and 2.9. The efficiency of each mesoionic derivative to inhibit the efflux of fluorescent substrates was determined by flow cytometry, relatively to controls (either parental cells, or the same transfected cells fully inhibited with reference inhibitors). The EC_50_ values (μM) were determined by using increasing derivatives concentrations, up to 50 μM, and calculated as derivatives concentrations producing half-maximal inhibition of drug efflux. The IG_50_ values (μM) were obtained by MTT assays upon treatment for 72 h with mesoionic derivatives at 0–100 μM; they were calculated as derivatives concentrations producing half-maximal inhibition of growth.

^a^IG_50_ obtained with resistant transfected cells

^b^IG_50_obtained with control, sensitive, cells; *n*.*d*. not determined

The same parameters were evaluated for the two other multidrug transporters, ABCG2 and MRP1. All derivatives significantly inhibited the ABCG2-mediated efflux of mitoxantrone: the extent observed at 25 μM after 30-min incubation was higher with MI-D containing a NO_2_ (~ 37%), but the affinity appeared better for MI-J containing a OH (EC_50_ value of 8.5 μM *versus* 24.3 μM). The two fluorinated derivatives (MI-4F and MI-2,4diF) displayed a lower inhibition (11.7–17.7%). No apparent transport by ABCG2 was observed, except for a weak possible effect of MI-J, with a RR value slightly > 1. No cross resistance at all was observed with MRP1-overexpressing cells, with RR values very close to unity. The different derivatives also inhibited MRP1-mediated drug efflux, using calcein-AM as a substrate, but with different structure-activity relationships when compared to ABCG2 since OH substitution in MI-J was much more efficient than NO_2_ in MI-D (36.2% *versus* 8.1% inhibition) while the fluorinated derivatives MI-4F and MI-2,4diF displayed intermediate potency (10–20% inhibition).

## Discussion and Conclusions

The present work reports a small series of new compounds as promising candidates for future assays of HCC treatment. The different mesoionic derivatives were cytotoxic to HepG2 cells, as demonstrated by MTT assays, with MI-J, MI-4F and MI-2,4diF being the most efficient to reduce their viability whereas MI-D required a 2-fold higher concentration. These results were confirmed by an increase in LDH activity of cell culture supernatants induced by all derivatives, with however some quantitative differences. The survival of non-tumoral hepatocytes in the presence of mesoionic derivatives demonstrated that MI-D, MI-J and MI-4F were not cytotoxic for these cells. By difference, a significant apparent cytotoxicity was observed with MI-2,4diF, in MTT assays, but no increase was produced on LDH activity. The absence of cytotoxicity was further confirmed by the lack of labeling with annexin V or PI, and by morphological analysis. Pires *et al*. [30] demonstrated that the alterations produced by 1,3,4-thiadiazolium derivatives on mitochondrial bioenergetics were associated with their hydrophobic properties: MI-2,4diF displayed the most pronounced effects, probably due to its high Hansh constant (π = 0,28) as compared to MI-4F, MI-D and MI-J. Therefore, the significant reduction of non-tumoral cell viability observed with MI-4diF in the MTT assay, which is based on the activity of mitochondrial dehydrogenases [50], might be related to its effects on mitochondrial bioenergetics. Induction of apoptosis by chemotherapeutics is one of the most significant effects related to inhibition of tumor growth [51]. When HepG2 cells were labeled here with annexin V and PI to monitor such an event, all mesoionic derivatives were indeed able to induce a significant double labeling. Annexin V is known to specifically bind to phosphatidylserine, a lipid which is translocated to the outer leaflet of the cell during apoptosis and can be identified through FITC fluorescence associated to annexin V [52]. PI is impermeable to cell membrane and its binding to DNA is dependent on the increased membrane permeability observed in the late stages of apoptosis or necrosis [53]. The results obtained with mesoionic compounds indicated that they induced a late apoptosis or necrosis in HepG2 cells. In order to better characterize the cytotoxicity pathway promoted, the HepG2 cells were incubated with the mesoionic derivatives, and the morphological analyses in earliest times of incubation indicated apoptosis features. These data were also confirmed by DNA fragmentation assays. Similar morphological alterations on melanoma cells MEL-85 were demonstrated with MI-D treatment (25–50μM for 2 h), which also reduced the viability of the cells to ~ 40% at 25 μM for 24 h [29]. In this work, we used primary culture of rat hepatocytes instead human cells due the scarce availability of fresh human liver samples, the logistic and time required by the overall procedure, as well as the high cost related to the procedure. As performed in this work, other studies [54–56] also have used rat primary hepatocytes in culture as an alternative to verify differential cytotoxicity of antitumoral compounds on human cancer. Nevertheless, the absence of cytotoxicity in rat cells observed in this work must be further confirmed on human cells. For new drugs intended to be used in clinical trials, several absorption, distribution, metabolism, excretion and toxicity assays are required, and the FDA has recommended initial *in vitro* tests to establish the effects of these drug candidates on MDR transporters, which could either promote their efflux or be inhibited by them, thus changing the bioavailability of other drugs used concomitantly [57]. We experimentally observed that mesoionic derivatives were not substrates of ABCG2 and MRP1, whereas they might indeed be transported by Pgp (except for MI-J), which could limit their use against resistant tumors overexpressing this efflux pump. This however would not prevent their utilization in nonresistant tumor treatment: for example, the chemotherapeutic agent 5-fluoracil is recognized as a first-choice treatment for nonresistant HCC in advanced stage [58], although displaying a high RR value of 53 on Pgp-overexpressing resistant HepG2 cells [59]. Some drugs with antitumoral and pump-efflux inhibitory activities, as also observed here for most 1,3,4-thiadiazolium derivatives, have indeed given promising results *in vivo*. As an example, the tyrosine kinase inhibitor BIBF120, which has reached phase III clinical trials of cancers treatments, also demonstrated a capacity to inhibit the ABCG2 transporter; however, the direct correlation between such an inhibition and the success of resistant-tumor treatment was not actually established [60]. The weak extent of inhibition by 1,3,4-thiadiazolium derivatives might still represent an advantage, taking into account the reduced probability of bioavailability alterations in polytherapy, and of diminution in the physiological protective role of these efflux pumps [61]. In conclusion, we showed that the 1,3,4-thiadiazolium derivatives MI-D, MI-J, MI-4F and MI-2,4diF were selectively cytotoxic to HepG2 cells, by promoting cell death with apoptosis characteristics, while not affecting the viability of non-tumoral hepatocytes. Furthermore, the 1,3,4-thiadiazolium derivatives were only slightly, or not at all, transported by resistant cells overexpressing ABCG2 and MRP1, while they even produced inhibition of these transporters. Such mesoionic compounds, especially the hydroxy derivative MI-J, might be considered as promising candidates to HCC treatment, either resistant or not, and should encourage new investigations about their mechanisms of action for future clinical tests.
